# 3D Printed Multiphasic Scaffolds for Osteochondral Repair: Challenges and Opportunities

**DOI:** 10.3390/ijms222212420

**Published:** 2021-11-17

**Authors:** Stephanie E. Doyle, Finn Snow, Serena Duchi, Cathal D. O’Connell, Carmine Onofrillo, Claudia Di Bella, Elena Pirogova

**Affiliations:** 1 Electrical and Biomedical Engineering, School of Engineering, RMIT University, Melbourne, VIC 3000, Australia; s3718367@student.rmit.edu.au (F.S.);; 2 ACMD, St Vincent’s Hospital Melbourne, Fitzroy, VIC 3065, Australia; serena.duchi@unimelb.edu.au (S.D.); carmine.onofrillo@unimelb.edu.au (C.O.); claudia.dibella@unimelb.edu.au (C.D.B.); 3 Department of Surgery, The University of Melbourne, St Vincent’s Hospital Melbourne, Fitzroy, VIC 3065, Australia; 4 ARC Centre of Excellence for Electromaterials Science, Intelligent Polymer Research Institute, University of Wollongong, Wollongong, NSW 2522, Australia; 5 Department of Orthopaedics, St Vincent’s Hospital Melbourne, Fitzroy, VIC 3065, Australia

**Keywords:** osteochondral, articular cartilage, calcified cartilage, subchondral bone, multiphasic, biofabrication, 3D printing

## Abstract

Osteochondral (OC) defects are debilitating joint injuries characterized by the loss of full thickness articular cartilage along with the underlying calcified cartilage through to the subchondral bone. While current surgical treatments can provide some relief from pain, none can fully repair all the components of the OC unit and restore its native function. Engineering OC tissue is challenging due to the presence of the three distinct tissue regions. Recent advances in additive manufacturing provide unprecedented control over the internal microstructure of bioscaffolds, the patterning of growth factors and the encapsulation of potentially regenerative cells. These developments are ushering in a new paradigm of ‘multiphasic’ scaffold designs in which the optimal micro-environment for each tissue region is individually crafted. Although the adoption of these techniques provides new opportunities in OC research, it also introduces challenges, such as creating tissue interfaces, integrating multiple fabrication techniques and co-culturing different cells within the same construct. This review captures the considerations and capabilities in developing 3D printed OC scaffolds, including materials, fabrication techniques, mechanical function, biological components and design.

## 1. Introduction

The structure of the knee allows for mobility and load-bearing movements [1]. Within the joint, the bone surfaces are covered with smooth, continuous articular (hyaline) cartilage which assists in movement and distribution of load [2]. Articular cartilage lacks vascularization and has a low cell density which contribute to its very poor ability to self-repair, implying that damage to the cartilage (chondral defects) will remain impaired and unfilled over time [3,4,5,6,7]. In contrast, injuries that include the underlying subchondral bone may be filled with fibrocartilage if the vascularization system in the bone is damaged causing the release of cells and factors which trigger a repair response [5,7,8,9]. Chondral defects can occur due to trauma or surgery or can arise idiopathically through general wear and tear during aging. Such defects are graded according to severity, each with their own treatment or pain-management strategies. OC defects are among the most severe and debilitating cases and are characterized by the complete loss of cartilage (articular and calcified) down to the underlying subchondral bone. OC defects have been detected in up to 20.8% of knee arthroscopies [10,11,12,13,14,15]. Without the ability to fully self-repair, empty OC defects can alter how forces are distributed over the joint and the areas where stress is concentrated [16]. Peak stress concentration can in turn accelerate the breakdown of the tissue surrounding the defect, leading to osteoarthritis [16].

Current surgical treatments for a full OC defect are limited and include microfracture or transplantation of OC tissue (allograft or autograft) [17,18]. While these treatments can provide some initial relief from pain, they have shown significant limitations, thus motivating research into regenerative approaches targeting full restoration of the damaged tissues as a long-term solution [19,20].

Tissue engineering strategies aim to restore tissue function using methods which combine cells, tissue-inducing agents (such as growth factors) and a scaffold (typically a biomaterial structure designed to guide regeneration) [21]. Cartilage and bone tissue engineering has been pursued since the 1990s, with most developed approaches based on highly simplistic, monolithic representations of each tissue type. In recent years, additive fabrication technologies have emerged, providing new tools for engineering living tissues, including bone and cartilage [22,23]. This adoption of additive fabrication techniques for the regeneration of living tissues has created the new subfield termed ‘biofabrication’ [24]. Biofabrication strategies offer enhanced control over the microstructural environment of engineered tissue by modulating the material(s), the structural design and the distribution of biological components. Such capabilities are especially relevant for recapitulating tissue interfaces, including the OC unit, since each respective tissue can be tailored to achieve the specific architectural framework and bioactivity [25].

With this rapid evolution of technology, recent reviews of OC regeneration have surveyed various specific aspects, including material focused; hydrogel-based 3D printed scaffolds, bioactive composite scaffolds, and fabrication method focused; scaffolds made via solid free-form techniques, printability requirements for OC bioinks [22,26,27,28]. In contrast, this review focuses holistically on recent advances in multiphasic additive manufactured OC scaffolds, including their main constitutive elements, i.e., materials, fabrication method, mechanical function, biological components and design. The articles reviewed here are limited to those that target the repair of the entire OC unit and exclude those which only address one of the three tissues individually (e.g., chondral defects only).

First, we describe the composition of native OC tissue and introduce current treatment methods. We then describe recent advances in engineering the OC tissue, with a particular focus on additive fabrication techniques. We next describe the methods for evaluating engineered OC tissue in vitro and in vivo, and finally conclude with a broader assessment of the field and future outlook.

## 2. Osteochondral Tissue: Anatomy, Pathology and Treatments

### 2.1. Structure of Osteochondral Tissue

**Figure 1:**
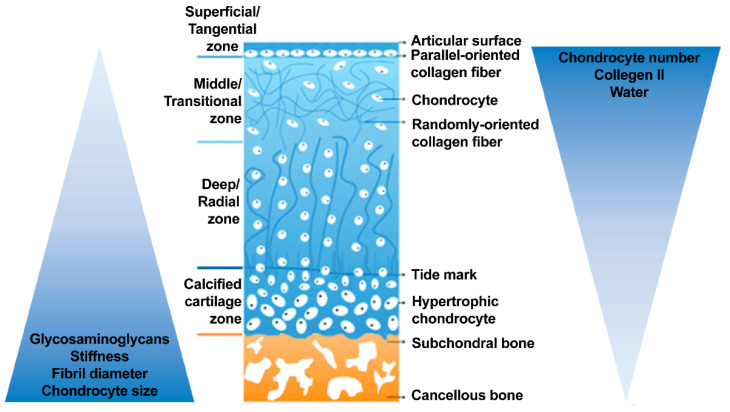
Gradient schematic of the OC tissue [31,32]. While the OC tissue is made up of articular cartilage, calcified cartilage and subchondral bone, each of these tissues are not homogenous. Especially in the articular cartilage, each zone within varies in cell size, number and orientation as well as collagen fiber size and orientation. Overall trends of the full OC unit are summarized in the ascending and descending triangles.

The native OC tissue consists of three different regions: the articular cartilage, the subchondral bone and the calcified cartilage-the interface or transition region in between. The three distinct regions have differing material compositions, fiber orientations and cell populations (Figure 1), and mechanical properties (Table 1). The articular cartilage can be further divided into the superficial, middle and deep zones, each with its own idiosyncratic microstructure determined by the extracellular matrix (ECM) structure and composition; chondrocyte number, shape and orientation; collagen type, and proteoglycan orientation (Figure 1) [29,30].

#### 2.1.1. Articular Cartilage

The average thickness of human articular cartilage varies depending on the site within the joint and the age of the patient. Hunziker et al. found the average human articular cartilage to be 2.41 ± 0.53 mm, but patients with joint diseases can display a decrease in thickness down to 1.48 ± 0.075 mm [36,41]. Despite being broken down into zones, the articular cartilage is a continuous region described as a ‘porous composite organic solid matrix swollen by water’ [42]. The mesoporous tissue has an average pore size of 6 nm which allows free water to move [42].

The outermost surface of the joint, the superficial or tangential zone, has the role of distributing loads evenly across its surface [43]. The chondrocytes residing in this zone are relatively small, flat and collagen fibrils are arranged parallel to the articular surface (Figure 1) [44,45]. Furthermore, the superficial zone has the highest deformation capability (out of three zones) and therefore is able to exchange fluids with the neighboring environment to better respond to compressive forces applied to the joint [45]. The middle or transitional zone provides some resistance to compressive force; the collagen fibrils contained in this area are not clearly orientated; proteoglycan content is the highest and chondrocytes are large and spherical in a loose columnar arrangement (Figure 1) [2,45,46,47,48]. The deep or radial zone provides the tissue with its greatest ability to withstand compressive forces due to collagen fibrils which run perpendicular to the cartilage surface [2,45,49,50]. The chondrocytes here are also arranged in perpendicular stacks (Figure 1) [43]. Water content is the lowest, and permeability is also lower compared with the superficial zones [45,51].

#### 2.1.2. Calcified Zone and Tidemark: The Transition/Interface

The calcified zone is made up of ≈22% unmineralized tissue which contains porous structures and then ≈88% mineralized tissue and, along with the subchondral bone, assists with shock absorption in the joint [52,53,54]. A few chondrocytes present in the calcified zone have limited metabolic activity and synthesize collagen type X, which can calcify the ECM [55]. The collagen fibrils run from the deep zone through the tidemark and calcified zone before anchoring to the subchondral bone with an orientation perpendicular to the tidemark [29,30,53]. The tidemark is a distinguishable line within the calcified zone which separates the calcified from the uncalcified articular cartilage with few to no cells [30,56,57]. The transitional property of calcified cartilage can be seen in its composition, at 65.1% hydroxyapatite (HA) dry weight for the calcified layer compared to 0% and 85.8%, respectively, for articular cartilage and subchondral bone [53,58].

#### 2.1.3. Subchondral Bone

The subchondral bone provides support by maintaining the joint shape, resisting stress and providing shock absorption as well as delivering nutrients through its vascular network [59,60]. The vascular structure in bone provides some limited regenerative capabilities as immune response cells and a cocktail of cells (osteoblasts, osteoclasts, osteocytes, chondrocytes, endothelial and mesenchymal stem/stromal cells (MSCs)) are present in this tissue and can be delivered directly to the area of impact [30,53,61]. Despite many cell types present in the subchondral bone, osteocytes are the most prominent (90–95% of total cells in the region) and are responsible for controlling signals to osteoblasts (bone formation) and osteoclasts (bone resorption) [53,62]. As a form of spongy bone, subchondral bone exhibits a low bone volume fraction of 6–36% resulting in a high porosity [63]. With some varying definitions of the term ‘subchondral bone’, in this review, we refer to subchondral as the region under the calcified cartilage [64].

### 2.2. Existing Surgical Treatments for Osteochondral Defects

**Figure 2:**
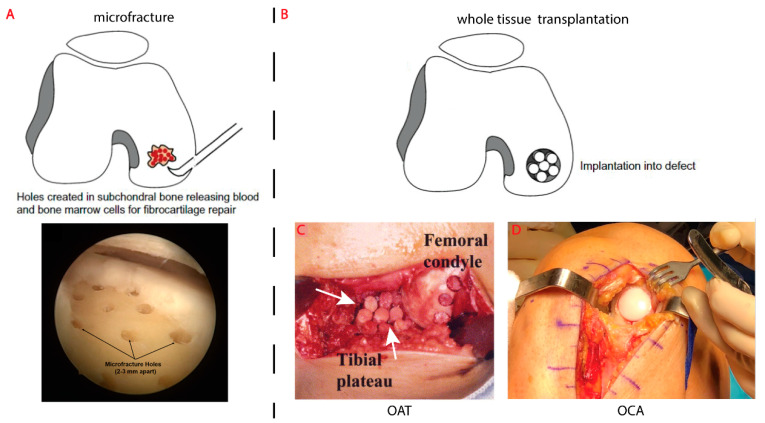
Current surgical techniques for the repair of an OC defect. (**A**). Microfracture [88] facilitate the repair of new tissue but primarily consists of fibrocartilage which fails to mimic the function of the native tissue [89]. (**B**). Whole tissue transplantation [88] of the mature OC unit can be from a patient (OAT) (**C**) [77] or from a donor (OCA) (**D**) [79]. The multiple plugs commonly used in OAT leaves gaps between plugs as well as between the plug and the host tissue (white arrows).

There are currently limited surgical options to repair an isolated OC defect [65,66]. In contrast, chondral-only defects have more surgical options available, including the tissue engineering techniques of autologous chondrocyte implantation and matrix-induced autologous chondrocyte implantation. However, these techniques are only focused on the repair/regeneration of the cartilage, while defects greater than 6–8 mm in depth (OC defects) require the addition of a bone graft [18]. Instead, relatively small OC defects, <2 cm^2^, can be treated using microfracture surgery where holes are created in the subchondral bone to access the bone marrow and release stem cells and growth factors (Figure 2A) [17,67,68,69,70]. While this method can produce new cartilage, histological analysis shows this to be mostly composed of fibrocartilage [70] which lacks the lubricative and load distribution properties typical of the native articular hyaline cartilage [54].

The conventional approach for the repair of OC defects is whole tissue transplantation (Figure 2B) [18], either as an autograft or allograft.

Osteochondral autograft transfer (OAT), also known as mosaicplasty, involves the transfer of OC cylindrical plugs from a non-weight bearing region of the patient’s joint to the defect (Figure 2C) [71,72,73,74,75]. While OAT can relieve pain in many patients, [76] the results are considerably less satisfactory when the defect is outside of a specified size range (typically 1–4 cm^2^ is recommended) or for patients over 35 [77,78,79,80,81]. One issue is how gaps between the plug(s) and the host cartilage can allow inflamed synovial fluid to penetrate and limit the healing and integration of the graft [82,83].

Osteochondral allograft transplants (OCA) involve transplanting an OC graft from a donor (bone bank) and is recommended for larger defects, greater than 2–4 cm^2^ (Figure 2D) [79,80,84]. As with OAT, the plugs of native tissue consist of all three OC tissues: articular cartilage, calcified cartilage and subchondral bone, with an overall height of the plug to match that of the defect. While OAT will often require multiple smaller plugs to fill a defect, OCA generally uses a single, larger plug, cut to size to match the defect shape [79]. However, issues with variability in tissue preparation and storage, infection, allograft/host mismatch, implant failure and arthritic degenerative changes, remain a significant limitation of this technique [85,86]. The risk of implant failure also increases for patients over 35 years old or who are female [86,87].

Of these existing treatments, only microfracture can facilitate new tissue growth, however, it does not produce the required hyaline-type cartilage. The average OC defect size reported in the literature is 4.1 cm^2^, which is above the recommended size for an OAT procedure [77]. The lack of an existing treatment with long term-effectiveness, particularly for larger OC defects, drives research towards a tissue engineered solution.

## 3. Engineering New Osteochondral Tissue

**Figure 3:**
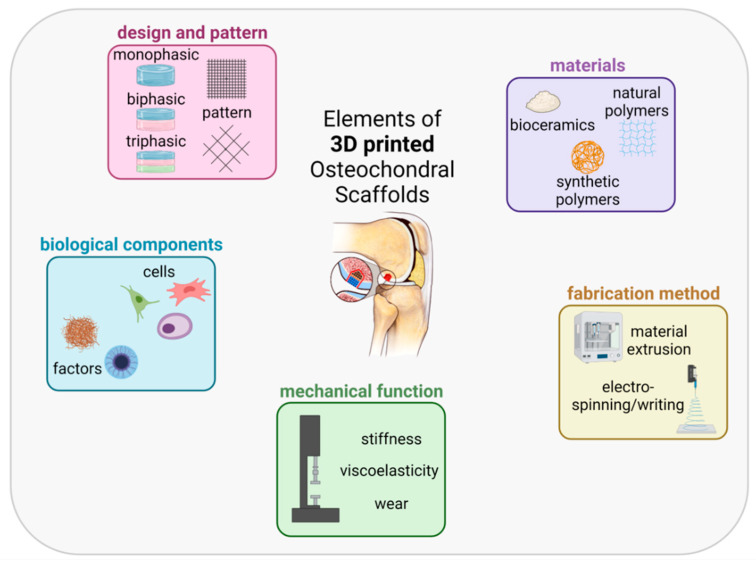
Elements of 3D printed OC scaffolds. We have identified five main domains of a 3D printed OC scaffold: materials (e.g., natural and synthetic polymers, bioceramics and hydrogels), fabrication method (e.g., material extrusion (ME), electro-spinning (ES), electrowriting and stereolithography (SLA), mechanical function, biological components (e.g., cells–chondrocytes, osteoblasts, stem cells and growth factors) the overall design (e.g., mono or multiphasic) and pattern of each 3D printed layer. Figure created with BioRender.com (10 October 2021).

The aim of an OC scaffold is to trigger the growth of new tissue in all its aspects, therefore mimicking the characteristics of the articular cartilage, calcified cartilage and subchondral bone. The scaffold must have the following functions: create a biocompatible environment, possess the mechanical function close to the native tissue, provide differential biological stimuli to address the lineage specific differentiation of implanted cells and production of the desired ECM, and possess the ability to integrate with the host tissue. Considering these requirements, we identified the following essential elements for the generation of the OC scaffold: material/s, fabrication method, mechanical function, biological component and scaffold design (Figure 3).

### 3.1. Elements of an OC Scaffold: Materials

Numerous scaffold criteria, such as biodegradability, processability, osteoinductivity, facilitating chondrogenesis, biocompatibilty and mechanical suitability can be adequately met via the correct selection of material(s) [90]. The materials that satisfy these criteria and are commonly utilized in OC scaffolds include natural and synthetic polymers and bioceramics, either alone or in combination [91].

#### 3.1.1. Natural Polymers

In OC engineering, natural polymers are commonly used in hydrogel form where their polymer networks are capable of holding a large amount of water thereby creating a fully hydrated 3D environment, comparable to that of the natural ECM [92,93]. This environment can support cell adhesion, proliferation and differentiation of various cells [94]. On the other hand, natural polymers typically possess weak mechanical properties, which can lead to deformation in weight bearing areas [95].

The base polymers processed from natural sources and widely used in OC scaffolds are gelatin, alginates, collagens and hyaluronic acid [96,97,98,99,100,101,102,103,104,105,106,107,108,109,110]. Of note, these natural materials are commonly modified, especially to allow for crosslinking (e.g., gelatin to gelatin methacryloyl (GelMA), hyaluronic acid to hyaluronic acid methacrylate) and as such become categorized as semi-synthetic materials [111,112]. Hyaluronic acid is typically used in the cartilage phase as it is a main component of the native cartilage ECM [102]. The weak mechanical properties are regularly emphasized as well as strategies to overcome this limitation include combining with another class of material or a reinforcement structure [96,98,105,108,110,113].

#### 3.1.2. Synthetic Polymers

Key advantages of synthetic polymers are enhanced mechanical properties (strength and stiffness) as well as their controllable biodegradability and processability [114]. However, synthetic polymers offer no specific biological influence over cells [115]. Polycaprolactone (PCL) is overwhelmingly used across all three phases of the OC scaffold, both on its own and when combined with other materials, due to its tunable biodegradability and approval by regulatory bodies, such as the Food and Goods Administration [98,99,101,103,104,105,106,110,116,117,118]. Polylactic acid (PLA) and poly(L-lactic-co-glycolic acid) (PLGA) are also used in all phases of the OC scaffold [104,119,120,121,122]. Critchley et al., compared the mechanical properties of PCL, PLA and PLGA (65:35 and 85:15 lactic acid to glycolic acid) in cartilage phase scaffolds [110]. After 21 days under physiological conditions, they found the PLGA 65:35, PLGA 85:15 and PLA had approximately a 956, 4.8 and 2.7 fold decrease in mechanical properties while the PCL did not change [110]. This finding highlights concerns of the suitability of PLGA in a load bearing joint due to its fast degradation rate and therefore rapid decrease in stiffness.

The hydrogel of synthetic origin, most commonly used in OC engineering, is polyethylene glycol (PEG) based, and these hydrogels are primarily used in the cartilage region of the scaffold [101,123,124,125]. PEG is an inert hydrogel providing a ‘blank canvas’ for modifying the degradation rate, mechanical properties and introducing biological drivers [125,126,127].

#### 3.1.3. Bioceramics

The category of bioceramics includes bioactive ceramics, bioactive glasses and bioresorbable ceramics which are primarily used in the bone phase and, to a lesser extent, the calcified cartilage phase of an OC scaffold [128]. Calcium phosphate cements and pastes, HA and tricalcium phosphate (TCP) are the most commonly used bioceramics [97,98,99,102,103,104,105,108,109,113,123,129,130,131]. These materials naturally exist as a brittle powder, thereby limiting their ability to form free-standing porous structures on their own [132]. Each material, formulation, source, and synthesis method within the category have varying levels of osteointegration, biomineralization, osteoinduction and osteoconduction capabilities [132,133,134]. In the study comparing PCL composites with various bioceramics, decellularized bone matrix, a natural product, was found to be more osteo-inductive then its synthetic counterparts HA and TCP, but these natural products are not commonly used in OC engineering [135].

Any combination of these material classes can be used with one another in order to minimize the undesirable properties and maximize the favorable ones. The use of multiple materials, especially in multiphasic scaffolds, allows the native OC environment to be more closely biomimetic.

### 3.2. Elements of an OC Scaffold: Fabrication Method

Selection of the fabrication method and material selection are interconnected and codependent steps within the OC scaffold design process. For example, when selecting a material, capabilities and compatibility of the suitable fabrication method should be taken into consideration (and vice versa). A single fabrication method can be used to create the entire OC scaffold or different methods can be used for creating the different phases. The most widely used additive manufacturing techniques for creating 3D printed OC scaffolds are ME, Melt Electro-Writing (MEW), ES, SLA and Digital Light Processing (DLP) [136]. Of note, this review captures only the commonly used 3D printing methods; however new 3D printing techniques are constantly emerging, including cyrogenic 3D printing [137], powder-based printing [138,139], indirect printing [140,141,142], phase separation [121] and custom-built printers [143].

In the native tissue, the articular cartilage is continuous, with an average pore size of only 6 nm, calcified cartilage has pores within the unmineralized areas which accounted for ≈22% of the tissue and subchondral bone has an overall porosity of 64–94% [42,52,63]. Using the native tissue as a template can guide the selection or suitability of each fabrication technique for each region of the OC scaffold.

#### 3.2.1. Material Extrusion

ME involves moving material through a nozzle to deposit it onto a print bed first on the XY plane before stepping up in the Z plane and continuing in a layer-by-layer fashion. As such, in standard ME printing, all material deposited needs to be (at least partially) physically supported by either the previous layers, support material or support bath [144].

ME printing allows for a broad range of materials to be used, including thermopolymers, hydrogels and bioceramics, where each category, material or composite, requires fine tuning of printing parameters, such as temperature, extrusion pressure, print speed and crosslinking or gelation for hydrogels [145,146,147,148]. ME allows for creating relatively porous scaffolds that enable cell proliferation and tissue ingrowth [149,150]. Smaller pores (<0.1–0.3 mm) can assist with neocartilage formation, and larger pores (>0.3 mm) can facilitate cell and bone growth [15,16,17,18,19,20,21,22,23,24,25,26,27,28,29,30,31,32,33,34,35,36,37,38,39,40,41,42,43,44,45,46,47,48,49,50,51,52,53,54,55,56,57,58,59,60,61,62,63,64,65,66,67,68,69,70,71,72,73,74,75,76,77,78,79,80,81,82,83,84,85,86,87,88,89,90,91,92,93,94,95,96,97,98,99,100,101,102,103,104,105,106,107,108,109,110,111,112,113,114,115,116,117,118,119,120,121,122,123,124,125,126,127,128,129,130,131,132,133,134,135,136,137,138,139,140,141,142,143,144,145,146,147,148,149,150,151,152,153].

**Figure 4:**
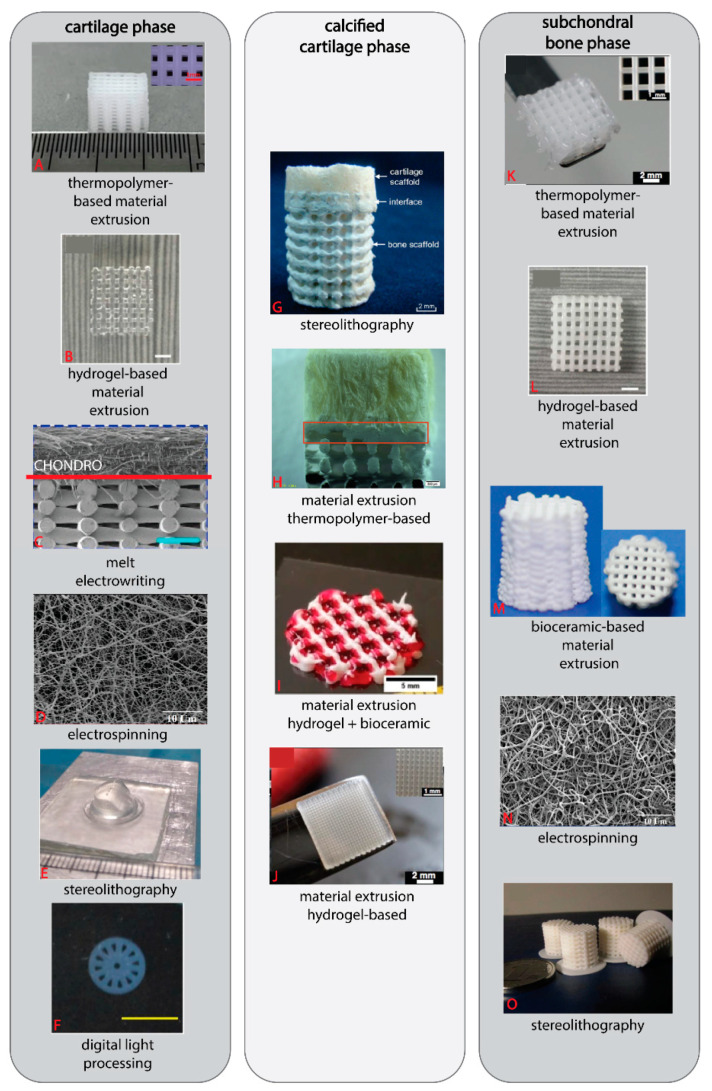
OC scaffold phases made via various 3D printing techniques. Some techniques, such as ME printing for thermopolymers and hydrogels, are commonly used across all phases of the OC scaffold, while other techniques, such as MEW, are commonly used only in one or two phases (cartilage and calcified cartilage). (**A**) Insert scale bar = 1 mm [183]. (**B**) Scale bar = 2 mm [113]. (**C**) Scale bar = 1 mm [184]. (**D**) Scale bar = 0.01 mm [165]. (**E**) [123]. (**F**) Scale bar = 5 mm [103]. (**G**) Scale bar = 2 mm [181]. (**H**) Scale bar = 0.5 mm [143]. (**I**) Scale bar = 5 mm [109]. (**J**) Scale bar = 2 mm, insert scale bar = 1 mm [104]. (**K**) Scale bar = 2 mm, insert scale bar = 1 mm [104]. (**L**) Scale bar = 2 mm [113]. (**M**) [156]. (**N**) Scale bar = 0.01 mm [165]. (**O**) [123].

The capabilities of thermopolymer-based ME (Figure 4A,H,K) are in creating porous structures which is most relevant to the subchondral bone phase. In the subchondral bone phase of multiphasic OC scaffolds, pore size is widespread between 0.3–1.0 mm, while the porosities lie between approximately 70–80% [103,106,130,154]. These indicative capabilities of ME extrusion with thermopolymer-based materials (the porosity range of subchondral bone and the pore size) were suggested to facilitate bone growth.

Bioceramic-based ME (Figure 4I,M) is also primarily used for the subchondral phase of the OC scaffold [105,109,155,156,157]. Overall, achievable porosities are lower in bioceramic printed scaffolds (≈20–60%) then thermopolymer printed ones (≈70–80%), which highlights the difficulties in creating high-porosity scaffolds with bioceramics-based materials. Pore size is also reduced (0.1–0.4 mm), with many scaffolds failing to produce the >0.3 mm pore size to facilitate bone growth in the subchondral bone phase [105,155,156]. Therefore, while the technique is widely used to create the subchondral bone phase of the OC scaffold, the ramifications of low porosity and pore size on tissue growth are not often displayed.

Hydrogel-based scaffolds in OC engineering (Figure 4B,I,J,L) are more commonly produced by simple casting techniques, especially on top of a 3D printed phase for the cartilage phase, and less commonly 3D printed to provide a specific architecture. This is likely due to the desire to mimic the continuous nature of the native articular cartilage. In addition, when employing biomimicry, physical characterizations such as such as the macro, meso and micro-porosity of the hydrogel are more relevant than pore size and porosity because these measurements are not used to describe the native tissue [103,158,159]. Kilian et al., produced the intermediate result between casting and 3D printing approaches by printing their hydrogel cartilage phase in a strand-wise pattern, thereby achieving a continuous layer [109]. The internal pore size depends largely on the material and is ranged between 100–800 nm and 100–220 µm, for alginate-based and GelMA 3D printed cartilage phases, respectively [103,108]. While larger than the pore size of native cartilage, the large pores can facilitate the distribution of nutrients, oxygen, the removal of waste and formation of neocartilage [103,108,151]. Despite hydrogel ME printed structures being commonly used in the cartilage phase, Gao et al., produced a biphasic OC scaffold entirely from hydrogel-based ME printing [113]. The subchondral bone phase included β-TCP in the base hydrogel which increased the stiffness and osteoinductive properties of the printed hydrogel, while the cartilage phase included transforming growth factor (TGF)-β1 to enhance chondrogenic differentiation [113].

#### 3.2.2. Melt Electro-Writing and Electrospinning 

MEW and ES also involve passing filament through a nozzle in a layer-by-layer deposition. However, in these techniques, voltage is used to control and continuously draw the filament onto the collector bed [160]. While fiber sizes range from the micrometer to nanometer scale, in practice MEW routinely produces micrometer fibers, while ES regularly produces nanometer fibers [161,162]. Furthermore, ES is a solvent-based technique that randomly deposits lines of material onto the collector bed; whereas MEW is a solvent-free approach that controls where and how the fibers are deposited, therefore providing control over the resulting pattern [162]. For MEW and ES, the material choice is driven by those that are processible following the electrohydrodynamic principles that guide both techniques [163,164]. PCL remains the most widely used material for MEW, while a broader range of materials, including PCL, gelation, chitosan, polyvinyl alcohol (PVA), HA and collagen are incorporated in ES [106,163,165,166]. Despite the increase in materials used in ES, the solvents used are typically toxic which can present a concern if toxic residues are left behind [167,168].

A challenge when applying MEW and ES to OC scaffolds is in creating tall 3D structures as a charge builds-up which limits the ability to have a stable jet of material in the Z direction [169,170]. This limitation, has been addressed to increase the height of produced structures by printing onto various collector beds and objects [164,169,171]. 

What does this mean for OC scaffolds? Given the limited height and micro/nano fibers produced, MEW and ES generally create softer scaffolds and thus have mainly been applied in creating the cartilage or the calcified cartilage phase (Figure 4C,D,N).

Highlighting the height limitations, Cui et al., applied ES to create a full, monophasic OC scaffold and while the 2–3 mm thickness was sufficient to fill a full OC defect in a rat model, this translates to the range of a chondral-only defect in humans [77,172]. Using these techniques in combination with other fabrication methods, printing onto non-flat collector beds or stacking scaffolds, Liu et al., and Hejazi et al., produced OC scaffolds at heights of ≈6 mm and 15 mm, respectively, which is suitable for the full repair of human OC defects [99,165].

#### 3.2.3. Stereolithography and Digital Light Processing 

SLA and DLP also create 3D objects by layer-by-layer material deposition. However, these techniques are not nozzle-based, and instead the liquid material sits in a resin bath where a build plate is lowered in and a light source traces the programmed pattern, crosslinking only the relevant design. The process continues the material deposition layer after layer until the object is completed.

The difference between SLA and DLP is the light source used. SLA utilizes a laser, while DLP uses light from a projector [173]. SLA/DLP can routinely print feature sizes of 50 µm, therefore placing the technique between MEW/ES and ME in terms of the resolution [174,175]. These techniques can be compatible with many of the same base materials previously outlined. However, extensive modification of the material is typically required which can greatly change the properties of said material [176,177,178,179]. Materials used in OC scaffolds include PEG based (commonly PEG-diacrylate), GelMA and TCP which are mixed with any combination of photoinitiators, photoabsorbers, solvents and/or dispersants [103,123,125,180,181]. In OC scaffold generation, SLA and DLP printing is not used as widely as ME, potentially due to the greater upfront and ongoing costs of these systems, limited biomaterials readily available or challenges in multi-material printing [180,182]. However, new research continues to emerge to overcome these issues, such as custom printers or new materials [182].

So far, SLA and DLP printed OC scaffolds (Figure 4E–G,O) have not been shown to offer advantages in scaffold porosity (≈50–65%) compared to the other techniques; however, Gong et al., used DLP to produce a radially orientated hydrogel cartilage phase which assisted with cell infiltration [103,125,181]. These techniques can also be used to create the entire OC scaffold as demonstrated by Zhu et al., who used DLP to create a monophasic OC scaffold from a PEG-based material combined with native bovine cartilage ECM [125].

### 3.3. Elements of an OC Scaffold: Mechanical Function

**Figure 5:**
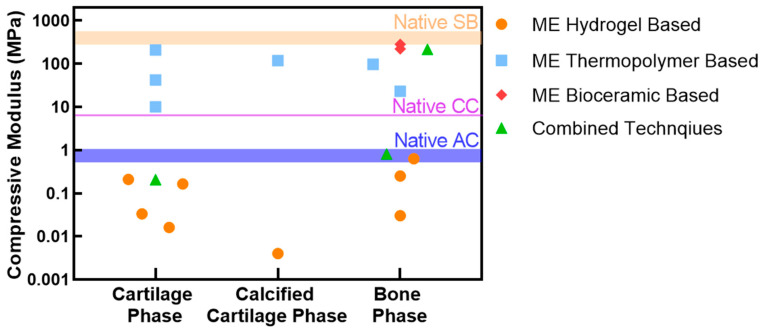
Compressive modulus of each phase of the OC scaffold. Data is limited for the calcified cartilage; however, the range of compressive modulus is similar regardless of the phase. Mechanical data for individual phases of the OC scaffold was also limited in techniques beyond ME. The bands represent the native tissue as from Table 1. Combined techniques refer to when multiple fabrication techniques are used to create the phase, e.g., ME thermopolymer scaffold filled with a hydrogel. Note: the band of the calcified cartilage (CC) is from indention and not compression of the whole tissue as for the articular cartilage (AC) and subchondral bone (SB). [96,97,99,104,113,121,183,193].

OC tissue primarily serves a mechanical, load-bearing function in the body. It is known that OC defects can change the distribution of forces across the joint, particularly for defects ≥10 mm in diameter [16]. Stress can concentrate near the rim of the defect, allowing the defect to grow in size if left untreated. The ultimate purpose of a biofabricated implant is to restore this load-bearing function and prevent further degradation. Thus, the mechanical function of an OC implant may be a critical factor in determining the success or failure of the intervention–especially in the immediate months after implantation, where the repair scaffold has not yet been able to produce mature tissue. In time, the new mature tissue may take over the mechanical function of the implanted scaffold in degradable scaffolds. However, the immediate stiffness mismatch or inadequate tuning of the degradation profile of the scaffold to that of the production of mature tissue, can cause the implant to fail due to the increased foreign body reaction and inflammatory response or large differences in the way the implant and tissue respond to applied forces [185,186,187,188]. This is in contrast to strategies, such as internal fixation devices, which are non-degradable and designed to retain their function immediately and over the life of the device/treatment (that may include prolonged load bearing and full range of motion of the joint) [189]. As such, the potential stiffness mismatch from the time of implantation is still an issue of consideration. The properties of the native tissue (Table 1 and colored bands Figure 5) are dependent on the mechanical test type, however the elastic modulus of human articular cartilage and subchondral bone (under unconfined compression) are approximately 0.64–0.854 MPa and 297–475 MPa, respectively, while calcified cartilage is ≈6.44 MPa under indentation [34,35,36,38]. As discussed, the negative implications of the stiffness mismatch between the implant and native tissue, as well as large variations in stiffness between each native OC regions are further justifying the need for a multiphasic OC scaffold, where, among other properties, the mechanical function can be designed to address the specific requirements of each constitutive region.

In addition to the multiphasic scaffold, the appropriate selection of material and fabrication method can minimize the ultimate stiffness mismatch as seen in Figure 5, where hydrogels are suited for softer structures, while thermopolymer and ceramic based ME are suited for stiffer structures.

For monophasic scaffolds, Wei et al., created a thermopolymer-based scaffold with a porosity of 75–79% and compressive modulus of 216–234 MPa. Hu et al., developed a natural polymer-based scaffold, with a porosity of 85–87% and compressive modulus of 4.16–7.59 MPa [190,191]. However, these homogenous scaffolds do not possess the ability to capture the different mechanical properties of each region of the native tissue.

Some research outputs provide a clear discussion on whether their OC scaffolds reach their desired mechanical function. For example, Chen et al., created a monophasic, ceramic-based scaffold (37–61% porosity) with a compressive strength of 15–40 MPa which, as reported, was sufficient and comparable to cancellous bone (2–12 MPa) [156]. Wang et al., noted that a high compressive strength would be desirable to induce differentiation in MSCs and their biphasic, thermopolymer-based OC scaffold showed an elastic modulus of 1.05, 14.1 and ≈6 MPa for the cartilage, bone and combined scaffold, respectively [192]. The thermopolymer-based scaffold (21–57% porosity) by Bittner et al., aimed to match the mechanical function of the ‘native tissue’. The reported compressive modulus of 102 ± 7 MPa of their scaffold is within the range of human trabecular bone [193]. Each research group has stated to meet their mechanical property targets. However, the absolute values, if provided, for each target tissue, were greatly varied due to different reference sources as well as using values from the native human vs. animal tissue which resulted in large variability in outputs between groups (Figure 5). While it would be logical for the mechanical function and values of native human OC tissue to serve as the benchmark, this is often not the case.

### 3.4. Elements of an OC Scaffold: Biological Components

The biological aspects of an OC scaffold include the elements placed within the construct, such as cells and growth factors as well as the culture conditions.

#### 3.4.1. Considerations of Cell Type

The use of cells within an OC scaffold can be a homogeneous or heterogenous approach, with one or multiple cell types used. Cells specifically used in the cartilage and subchondral bone region include chondrocytes, chondroprogenitor cells and osteoblasts, while the most common cell type used across the entire OC scaffold are MSCs of different species and sources [100,102,105,106,109,194,195,196,197]. While MSCs are multipotent, induced pluripotent stem cells are, as the name suggests, pluripotent and therefore offer the ability to repair all the different OC tissue regions. However, to date this cell type has been rarely used in conjunction with 3D printed scaffolds for OC repair, potentially due to challenges in achieving uniform cell differentiation or highly variable results in chondrogenesis as well as increased regulatory hurdles with induced pluripotent stem cells [198,199,200,201]. Alternatively, the challenge with chondrocytes includes their ability to dedifferentiate and thereby stop or minimally produce collagen II which is the main component in hyaline cartilage [202,203]. In the development of OC scaffolds, both osteoblasts and chondrocytes are derived from the mesoderm, suggesting the potential to populate a multiphasic OC scaffold with a homogenous population of MSCs, which are then directed along alternate differentiation pathways using the tailored microenvironment within each phase. This strategy was employed by Liu et al., where bone marrow derived MSCs (bmMSCs) were present in each phase of the OC scaffold [102]. The scaffolds were subcutaneously implanted in rats, and after two months chondrogenic and osteogenic differentiation were achieved in the respective phases due to the targeted materials and controlled release of stem cell differentiation inducers [102]. This strategy presents new opportunities along with the complexities further discussed below.

#### 3.4.2. Culture Conditions and Growth Factors

Cells, grown in vitro, require specific conditions to grow and proliferate, let alone differentiate. This involves tuning conditions, such as oxygen content, media type (including the base media, serums and additional factors) and concentration, and regular media replacement [204]. The addition of physical cues, such as mechanical, electrical and electromagnetic, can influence cell signaling pathways and affect chondrogenesis or osteogenesis. Balancing all these conditions becomes even more challenging when multiple cell types are involved.

Growth factors also aim to influence cell behavior such as growth, proliferation and differentiation [205,206]. Growth factors, relevant to supporting neocartilage formation, include insulin-like growth factor (IGF) 1, bone morphogenetic proteins (BMP) 2 and 6, BMP-7, fibroblast growth factor (FGF) 2, platelet-derived growth factor and TGF-β1 and TGF-β3 [207,208,209,210,211,212,213,214,215,216]. Growth factors, relevant to subchondral bone formation, include BMP-2, BMP-7, FGF-2 and IGF-1 [217,218,219,220]. Growth factors have a short half-life, therefore need to be regularly added to the culture environment, such as in the media, for in vitro or ex vivo culture [221]. Relying on growth factors in the culture environment presents a challenge when simultaneously aiming to stimulate cell(s) down different lineages in the one scaffold. To address this challenge, sophisticated culture conditions and bioreactors can be used, allowing different phases of the scaffold to be in direct contact with different medias and their associated growth factors [222,223]. Otherwise, the growth factors can be incorporated into the OC scaffold for sustained release either via passive release, triggered release or encapsulation (chemical or physical) [221]. Modulating the timing of this release as well as the crosstalk between layers is crucial. However, this mechanism has not been fully elucidated yet for use in tissue engineered strategies [224].

### 3.5. Elements of an OC Scaffold: Design

**Figure 6:**
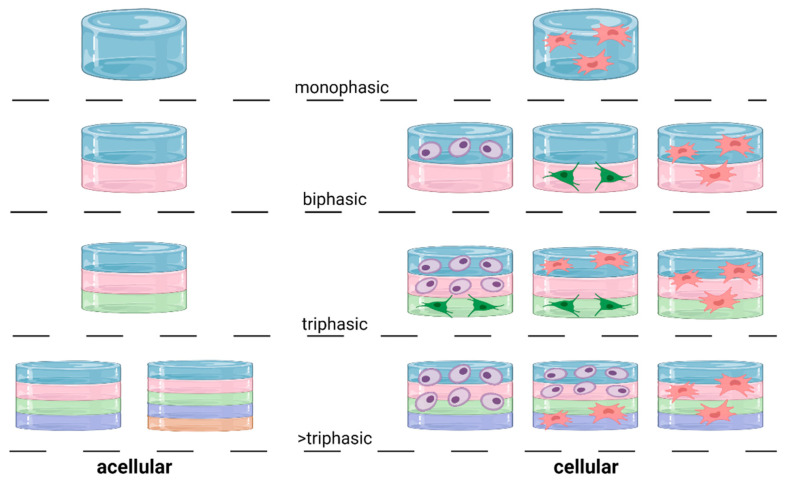
Design options for an OC scaffold. Categories include mono (one) phase, bi (two) phases, tri (three) phases and >tri (more than three phases). Any combination of number of phases, materials, and cell type can be used to create the ‘ideal’ OC scaffold. For example, chondrocytes (purple) are commonly used in the cartilage phase/s, osteoblasts (green) in the subchondral bone phase/s while MSCs (pink) are used throughout the whole OC scaffold. Figure created with BioRender.com (10 October 21).

The design of an OC scaffold can be split into a mono, bi, tri or >triphasic design. In theory, a monophasic scaffold cannot capture the specific properties of each tissue, whereas multiphasic scaffolds were shown to replicate some of the properties of at least two of the three tissues by synergizing different combinations of materials, biological and/or mechanical elements in each phase (Figure 6). However, increasing the number of phases can also increase the complexity of fabrication and analysis. One extra category of design is a gradient scaffold, where each phase blends into one another rather than a distinct separation [225]. This is more difficult to achieve through nozzle-based fabrication as a filament or material reservoir is generally filled with a homogenous material, and the changing of materials requires the switching of reservoirs which creates a distinct separation between phases. As such, gradient scaffolds are not in the scope of this review.

Another consideration in the design of a 3D printed OC scaffold is the printed pattern of each layer. This pattern affects the interconnectivity of pores as well as overall porosity. Creating this desired pattern can be limited by the capabilities of the fabrication technique. As reported, fully interconnected, porous scaffolds facilitate cell migration through the entire construct, the diffusion of nutrients and removal of waste [226,227].

#### 3.5.1. Monophasic Scaffold

**Figure 7:**
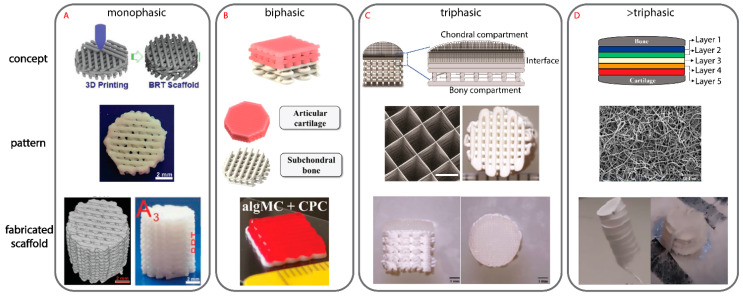
Examples of different scaffold design approaches including the pattern of each 3D printed layer. (**A**) A monophasic scaffold made from a homogenous ceramic paste ME printed. The text in the fabricated scaffold image is from the original research output [196]. (**B**) A biphasic scaffold with both phases ME printed. The subchondral bone phase is made from a ceramic paste and the cartilage phase is made from a hydrogel [109]. (**C**) A triphasic scaffold made from ME and MEW where the subchondral bone phase is made from a ceramic paste, the cartilage phase contains a thermopolymer MEW mesh and hydrogel while the interface contains all the forementioned materials [105]. (**D**) A >triphasic scaffold has more than three distinct phases with this example scaffold containing five individual phases all made from ES [165].

A monophasic scaffold (Figure 7A) relies on the homogeneous design and material (or material composite) to repair the entire OC defect. This translates to a single porosity and mechanical property of the scaffold required to withstand the relevant forces in each tissue region. However, the ability to repair different tissues in an OC defect using a monophasic scaffold is challenging. Monophasic OC scaffolds have been created using a range of materials and biofabrication techniques, including ES of zinc oxide-PCL composite and ME of bioceramic [196,228]. In both cases, either concentrations or surface features were altered in order achieve enhanced chondrogenic or osteogenic potential, but the monophasic scaffolds on their own cannot achieve both.

#### 3.5.2. Biphasic Scaffold

Here, a biphasic scaffold (Figure 7B) is referred to the targeted repair of the bone and cartilage phases, typically neglecting the calcified cartilage in-between. Splitting the scaffold into two allows customization in one or more elements, i.e., the material, design, porosity, mechanical function and/or cell type. This gives a greater ability to more closely mimic the structure of the natural tissue and drive the formation of multiple new tissues.

Kilian et al., characterized various biphasic combinations of their acellular ceramic ‘cement’ and cellular alginate-based hydrogel or by combining the two materials aimed to address the calcified cartilage [109]. The 3D printed scaffolds were confined to a 0, 90° log pile design that could have been designed or a limitation of the printing technique in combination with the materials. The biphasic scaffold by Critchley et al., featured a ME printed PCL scaffolds with a standard 0, 90° log pile design [110]. While utilizing a range of cells throughout the construct, the biphasic scaffold was cultured in chondrogenic media, therefore placing emphasis on the cellular response in the cartilage phase over that of the bone phase [110]. Despite this, after 6 months of the scaffolds implanted in vivo in an OC defect in a goat, the team observed the production of hyaline-like articular cartilage in most animals, but inconsistent results in the quality of subchondral bone were produced [110].

#### 3.5.3. Triphasic Scaffold

Developing a triphasic scaffold (Figure 7C) allows for the interface or calcified cartilage phase to also be prioritized. Key considerations in each phase of the scaffold include the material, design and which cells, if any, are used (Table 2).

#### 3.5.4. Triphasic Scaffold

The final category of design is the most complex with a minimum of four distinct phases (>triphasic therefore more than three phases) in the OC scaffold (Figure 7D). While this approach is not widely used, moving from three to four or more phases usually results in the breakdown of the cartilage phases into its different zones.

Mancini et al., have presented an OC scaffold consisting of four distinct phases, with the aim of better capturing the zonal nature of articular cartilage [101,229]. The PCL scaffold with a 0°, 90° log pile pattern serves as the base with the porosity gradually decreasing to a solid layer to act as the interface region. The log pile PCL then continues to a 70% porosity along and a hydrogel containing equine MSCs. The PCL is then removed leaving only hydrogel and MSCs followed by the final phase, with again the same hydrogel but with ACPCs instead of MSCs. The rationale for a stronger focus on the cartilage repair was due to its distinct lack of intrinsic healing capability without a vasculature structure [229].

Further increasing the number of phases is work reported by Hejazi et al., where a five-phase electrospun OC scaffold was developed [165]. The five-phase scaffold is based off various combinations of PCL, gelatin, chitosan, PVA and nano-HA. The team created the OC scaffold in the relevant height for an OC defect (15 mm); however, with no full biological characterization of the scaffold, it is yet to be seen whether the scaffold with five phases offers a benefit over those with 2–4 phases [165].

A representative scaffold from each of the four categories of deigns can be seen in Figure 7.

## 4. Functional Evaluation: In Vitro and In Vivo

The response of the scaffold under physiological conditions needs to be evaluated and can be achieved by using an in vitro, ex vivo, in vivo (animal models) and in vivo (human) studies.

An in vitro model is a logical first experiment to test the response of the OC scaffold as it is the cheapest, lowest risk and fastest way to generate biological results (Table 3) [230,231,232].

An ex vivo model can be a powerful tool lying between that of in vitro and in vivo models, where tissue from a human or animal is used outside of its original environment. A practical use of this model in OC research is to create an artificial defect in a condyle plug to implant in the tissue engineered scaffold. This model is useful for studying the integration between the implant and native tissue. However, the sample can be more difficult to post-process and analyze given multiple materials/tissues of varying compositions. Despite the potential of the ex vivo model, to date it has been rarely used to study a 3D printed full OC scaffold and is more commonly used in chondral-only defects [233,234,235,236,237,238].

For in vivo models, small models, such as rabbits and mice, are used more often than large animals-largely due to the reduced costs; however, the structure of the OC tissue is closer to humans in large models, such as horses, sheep and pigs and therefore be more beneficial in the regulatory pathway [101,239,240,241,242]. In addition, some small animals, especially rabbits, have a self-healing capability which means results from these models can be misleading [241]. These animal models, especially large models, are often a pre-clinical requirement and can provide significant information in predicting the fate of the treatment in humans (Table 4).

Human in vivo experiments or clinical trials are the ultimate model to achieve clinical translation. However, the road to human clinical trials is long with rigorous ethical, legal and documentation checkpoints as well as high costs. Unlike the previous models, an OC scaffold implant cannot be removed after a specific time period and instead, techniques including MRI and patient questionnaires are used. According to the Clinical Trials database by the U.S. National Library of Medicine (search was based on using the keywords ‘osteochondral’ and ‘3D print’), there are currently no clinical trials (underway, completed or recruiting) related to 3D printed scaffolds for OC repair in the knee. However, there have been a limited number of clinical trials related to tissue engineering OC scaffolds, not fabricated by 3D printing, including (i) ChondroMimetic, a biphasic scaffold with a cartilage phase made of collagen and GAG and a bone phase also made of collagen and GAG plus calcium phosphate (study completed); (ii) Agili-C™, a porous, resorbable biphasic scaffold, containing calcium carbonate (study completed) and BioMatrix CRD, a biphasic, bioresorbable scaffold containing collagen in the cartilage phase and TCP in the bone layer (study completed).

All the evaluation stages, from non-clinical, i.e., in vitro and animal models, to clinical trials are aligned with the regulatory framework established for medical devices [242].

## 5. Discussion and Future Outlooks

This review discusses in detail the approaches to creating scaffolds for the repair of the entire OC tissue, including the subchondral bone, articular cartilage and the calcified cartilage in between. We have presented five main areas of consideration when creating an OC scaffold, i.e., material, fabrication method, mechanical function, biological components and design. Each of these five requirements for creating the OC scaffold are heavily interconnected.

Considerations for the material include its physical properties, printability, biological response and inherit mechanical properties. Some of these properties, such as printability, may be enhanced through substantial optimization of the printing parameters or modification of the material [244]. Included in the multiphasic scaffold method, is a multi-material approach which offers the ability to carefully select the material for each specific region of the scaffold to elicit the desired response. While no single material has been proven to be superior to their counterparts, bioceramics, including HA and TCP, are specific to the subchondral bone phase, while hydrogels are becoming the material of choice for the cartilage phase. In current approaches, the interface region is often only a combination of the materials used in the cartilage and subchondral phase. This shows that the calcified cartilage is not understood as well as its adjacent tissues (articular cartilage and subchondral bone).

In more than 80 research studies, focused on developing 3D printed OC scaffolds and included in this review, ME printing was the most commonly used fabrication technique. This is likely due to ME printing’s wide availability, material versatility and low cost. While MEW and ES are still popular choices, they are primarily used for the cartilage phase only, and their traditional print style limits the achievable scaffold thickness. This results in the necessity of stacking many scaffolds or sheets aiming to reach a relevant or desired thickness. The use and choice of a fabrication method should not be underestimated as it primarily controls critical scaffold design parameters, such as pore size and porosity, which can facilitate tissue ingrowth. Using a combination of 3D printing techniques is common in the literature and offers a promising approach to better capture the varying tissue regions, especially in terms of mechanical functionality [105].

While there is evidence to suggest that the mechanical function of a biological implant should be close to that of the native tissue, there are still many research outputs that do not prioritize the mechanical characterization of their scaffolds [245,246,247]. Overall, compressive-based measurements featured more often than tensile or shear stress tests in the literature, suggesting that when the forces on the condyles of the knee are simplified, compression is seen to be dominant. The true importance of the mechanical function is likely not to be seen until long-term in vivo studies with a large-scale animal model are conducted, where the movement and effect of load-bearing is more similar to that of humans.

The element of design provides an insight to where the field is heading, with a continued emphasis on multiphasic scaffolds. Increasing the number of phases in a scaffold provides the ability to tailor each region to the specific tissue rather than trying to repair three tissues with a single material, pattern and/or cell type. Further into the overall design is the pattern of each 3D printed layer driven by the capabilities of the fabrication technique with a given material. The 0, 90° log pile continues to be the most common pattern in 3D printed OC scaffolds. This represents a limitation in nozzle-based fabrication, where each layer needs to be at least partially supported by itself or a support material in order to create a 3D structure [144]. While a 0, 90° log pile pattern can produce an overall interconnected scaffold, if there is good definition (i.e., fibers do not sag thereby touching the layers below) in each layer, alternative designs, including lattice structures, can offer a greater freedom in design pattern, porosity and mechanical function but are not commonly used in nozzle-based fabrication. While SLA approaches can circumvent this design limitation, there is little demonstration of this with OC scaffolds [123,125,181,248]. This offers a significant opportunity for future research in developing OC scaffolds.

The fully fabricated scaffold needs to be evaluated to test the combination of the core elements via biological characterization, including in vitro, ex vivo, in vivo (animal) and in vivo (human)/clinical trials. In this order the model increases in complexity, cost, resources and time of experiments but also represents a logical progression in the translation from bench to clinic. The ex vivo model offers an opportunity to study the integration of the OC scaffold with the native tissue, thus presenting another research direction in developing advanced OC scaffolds.

Finally, a significant challenge to address is the lack of standardization. This extends to the terminology used, type of mechanical test performed, output presented, biological characterization and markers used, etc. While the lack of standardization is common across all areas of tissue engineering, it makes it difficult to conclude which OC scaffold in the research stage is the most promising for translation to clinic. This is especially evident in the mechanical function, since despite the OC defect occurring in a weight bearing region, not all research outputs include any mechanical data nor discuss the implication on the design and function of the developed scaffold. Furthermore, even when there are shared targets between research studies, such as matching the mechanical function of the scaffold to the native tissue, that native tissue target can still vary depending on various factors, including if the target values are from native animal or human tissue, diseased or healthy tissue, type of test and test conditions etc. One way to help overcome this limitation could be to employ a streamlined mechanical protocol, like that produced by Kabir et al., which allows for the sequential testing of a single sample to generate many key pieces of relevant mechanical data rather than only one or two [33].

It is widely reported that the current surgical approaches for repairing an OC defect still have significant drawbacks, therefore a 3D printed multiphasic scaffold approach offers the potential to achieve a superior result by repairing each of the native tissues in the OC unit. The full capabilities of 3D printing are currently underutilized and thus, present an area for potential improvement in the design capabilities and biomimicry of the printed scaffolds. While there is no single material or fabrication method that has proven to be superior in developing an OC scaffold, a multiphasic scaffold seems to be the best approach to creating tailored environments to regrow each of the elements within the OC unit. The calcified cartilage region remains the least understood region in the tissue engineered scaffold, as such, the role of this region needs to be better defined and addressed. However, the strong focus on biofabricated multiphasic scaffolds provides an optimistic outlook to developing a method for full repair of the OC function and its further translation to an improved clinical treatment and patient outcome.

## Figures and Tables

**Table 1:** Compressive-based mechanical properties of the human OC tissue.

OC Region	Mechanical Test	Elastic/Young’s Modulus	Ref
Articular Cartilage	Indentation	1.03 ± 0.48 Mpa	[33]
	Unconfined compression	0.854 ± 0.348 MPa	[34]
		0.64 ± 0.30 MPa	[35]
Calcified Cartilage	Indentation	6.44 ± 1.02 MPa	[36]
Subchondral Bone	Indentation	≈6–13 GPa	[37]
	Unconfined compression	297–475 MPa	[38]
	Unconfined compression via finite element modelling	3–20 GPa	[39]
		296 ± 107–497 ± 52 MPa	[40]

**Table 2:** Triphasic OC scaffolds.

	Cartilage Phase	Calcified Cartilage Phase	Subchondral Bone Phase	Ref
Material	Methacrylated hyaluronan, isocynatoethyl acrylate−modified β−cyclodextrin, kartogenin	All materials found in cartilage and bone phase	HA, alendronate	[102]
Design	Homogenously casted hydrogel	0°, 90° log pile infiltrated with homogenously casted hydrogel	0°, 90° logpile	
Cells	Human bmMSCs *	bmMSCs *	Human bmMSCs *	
Material	Cartilage ECM, chitosan	PLGA, TCP	PLGA, TCP	[121]
Design	Orientated casted hydrogel	0°, 90° log pile however ≈ 50 µm spacing between fibers so practically close to a solid disk	0°, 90° logpile	
Cells	Goat bmMSCs	Acellular	Goat bmMSCs	
Material	Alginate, PLA	Alginate, GelMA, TCP,	PCL	[104]
Design	0°, 90° log pile	0°, 90° log pile	0°, 90° log pile	
Cells	Acellular	Acellular	Acellular	
Material	PCL, GelMA	PCL + all materials in cartilage phase	α-TCP, nano−HA, hydrogel (either unmodified or modified poloxamer)	[105]
Design	0°, 90° log pile PCL infiltrated with homogenously casted GelMA	0°, −0°, −90°, −90° log pile (cartilage phase) and 0°, 90° log pile (bone phase)	0°, −0°, −90°, −90° log pile	
Cells	Articular cartilage progenitor cells (ACPCs)	Acellular	Acellular	
Material	Sodium alginate	Sodium alginate, mesoporous bioactive glasses	Sodium alginate, mesoporous bioactive glasses	[107]
Design	0°, 90° log pile	Dense/solid phase	0°, 60° rotation steps	
Cells	Acellular	Acellular	Acellular	

* Cells were seeded/incorporated into the scaffold to assess cytocompatibility only and do not necessarily represent the whole/final strategy for repair.

**Table 3:** In vitro models for the analysis of OC scaffolds.

In Vitro					
Design	Materials	Elastic Modulus	Degradation	Outcome	Ref
Mono-phasic	Insulin, PLGA, polydopamine, PCL	Monophasic scaffold: 233.71 ± 7.57 MPa	N/A	Significant increase in cell number, alkaline phosphatase, glycosaminoglycan/protein and Alizarin Red after 7–14 days when MSCs and chondrocytes were seeded onto the scaffold. There was also significant increase in SOX-9, collagen I and aggrecan suggesting chondrogenic differentiation and RUNX-2, collagen II and osteocalcin suggesting osteogenic differentiation.	[190]
Biphasic	PLA, PCL, HA, chitosan, silk firoin	Cartilage phase: 1.01 ± 0.04 GPa Bone phase: 1.07 ± 0.16 GPa	0.33 ± 0.09% after 30 days	Cell viability increased from 125.25 ± 9.36% to 308.28 ± 7.88% from day 1 to 14 respectively. The presence of HA and CS/SF increased cell proliferation.	[119]
Biphasic	P(NAGA-co-THMMA) hydrogels, β-TCP	Biphasic scaffold: 16–115 kPa	N/A	Significant increase in collagen II and aggrecan after 14 days. Significant increase in alkaline phosphatase, collagen I, osteocalcin and RUNX2 after 14 days cultured in non-osteogenic media.	[113]
Biphasic	PCL, HA, interleukin-4 GelMA	Biphasic scaffold: 73 ± 1 to 75 ± 3 MPa	≈75% weight loss in 8 weeks	The cartilage scaffold was anti-inflammatory and had an increase in cell number after 5 days. Increase in RUNX2 and Alizarin Red staining in subchondral phase compared to the control.	[103]
Multi-phasic	PCL, PVA gelation, chitosan, nano-HA,	Multiphasic scaffold: 6.2 ± 0.5 MPa (low strain) 70 ± 29 MPa (40% strain)	≈35% weight loss in 12 weeks	Increase in MSC cell number over 21 days. Greater cell density, proliferation, and migration in the subchondral bone phase over the cartilage.	[165]

**Table 4:** In vivo animals models for OC scaffolds.

In Vivo (Animals)					
Animal	Design	Materials	Duration	Outcome	Ref
Rabbit	Monophasic	Self-assembling peptide hydrogel coated PCL	12 weeks	Coating with hydrogel reduces chondrocyte death rate, and enhanced cell growth. Highly improved hydrophilicity and biomimetic ECM structures. Promoted neobone and neocartilage regeneration.	[117]
Rabbit	Biphasic	mPEG-PCL, HA, glycidyl methacrylate-hyaluronic acid, TGF-β1	12 weeks	The empty control had neobone formation only while the scaffold group had neobone and neocarilage formation. Some scaffold remained in the defect.	[130]
Rat	Biphasic	P(NAGA-co-THMMA) hydrogels, β-TCP	12 weeks	In the subchondral bone phase there was a significant increase in the total volume of tissue regenerated and bone mineral density compared to the control group and there was strong staining for osteocalcin, collagen I and toluidine blue. Neocartilage formation was present in the cartilage region with strong staining for glycosaminoglycan, collagen II and toluidine blue.	[113]
Rabbit	Biphasic	PCL, HA, interleukin-4 GelMA	16 weeks	In the subchondral bone phase there was a significant increase in the total volume of tissue regenerated compared to the control group. Qualitative and quantitative Safranin O staining results were higher compared to the control.	[103]
Rabbit	Biphasic	β-TCP, PEG	12 months	By 12 months there was tissue formation the entire defect. In the subchondral bone phase there was a significant increase in the total volume of tissue regenerated at 24 weeks compared to the control.	[124]
Mini-pigs	Biphasic	mPEG-PCL, HA, glycidyl methacrylate-hyaluronic acid, TGF-β1	12 months	Scaffold was still present in the subchondral bone phase while the cartilage phase was taken over by semi-mature cartilage. The subchondral bone phase also contained mixed bone and fibrotic tissue Of note: the control defect was completely filled but with fibrocartilage.	[243]

## Data Availability

Not applicable.

## Abbreviations

OC	Osteochondral
OAT	Osteochondral Autograft Transfer
OCA	Osteochondral Allograft Transplant
PLA	Polylactic Acid
PCL	Polycaprolactone
PLGA	Poly(L-lactic-co-Glycolic Acid)
PEG	Polyethylene Glycol
PVA	Polyvinyl Alcohol
ECM	Extracellular Matrix
HA	Hydroxyapatite
TCP	Tricalcium Phosphate
ME	Material Extrusion
MEW	Melt Electro-Writing
ES	Electro-Spinning
SLA	Stereolithography
DLP	Digital Light Processing
MSC	Mesenchymal Stem/Stromal Cell
bmMSC	Bone Marrow derived Mesenchymal Stem/Stromal Cell
ACPC	Articular Cartilage Progenitor Cell
IGF	Insulin-like Growth Factor
BMP	Bone Morphogenetic Proteins
FGF	Fibroblast Growth Factor
TGF	Transforming Growth Factor
